# Senior–Loken Syndrome: Ocular Perspectives on Genetics, Pathogenesis, and Management

**DOI:** 10.3390/biom15050667

**Published:** 2025-05-05

**Authors:** Di Zhou, Yi Zeng, Weihan Luo, Chenyang Leng, Chen Li

**Affiliations:** 1 Center for Drug Evaluation, National Medical Products Administration, Beijing 100076, China; zhoud@cde.org.cn; 2 Xiangya School of Medicine, Central South University, Changsha 410013, China; 8301200110@csu.edu.cn (Y.Z.); weihanlo118@gmail.com (W.L.)

**Keywords:** Senior–Loken syndrome, nephronophthisis, ciliopathies, primary cilium, retinal photoreceptors, clinical features, pathogenesis, genetic basis, treatments

## Abstract

Senior–Loken syndrome (SLSN) is a group of rare autosomal recessive disorders caused by dysfunction of the primary cilium, primarily affecting the kidneys (typically leading to nephronophthisis) and eyes (typically leading to retinal degeneration). Moreover, patients with SLSN may experience additional multisystemic symptoms, such as developmental delay, intellectual disability, ataxia, and nystagmus. To date, eight genes have been demonstrated to cause SLSN, all encoding for proteins involved in the structure and functions of the primary cilium. This places SLSN within an expanding category of diseases known as “ciliopathies”. Due to the genetic heterogeneity and significant phenotypic overlap with other ciliopathies, establishing a definitive diagnosis during the initial consultation remains a challenge for clinicians. Furthermore, current research on SLSN-related ciliopathies predominantly focuses on renal involvement, while the ocular manifestations remain insufficiently explored and lack a comprehensive review. Therefore, with the goal of offering practical guidance for clinical practice, this review aims to provide a comprehensive overview of the clinical features, along with an ocular perspective on the molecular mechanisms, genetic underpinnings, and advances in the treatment of SLSN.

## 1. Introduction

Senior–Loken syndrome (SLSN) is a rare autosomal recessive disorder characterized by retinal degeneration and nephronophthisis (NPHP), affecting approximately 1 in every 1 million individuals [1,2]. The pathogenetic basis of SLSN involves the primary cilium, a highly conserved structure in cellular development. In retinal photoreceptors, it serves as a specialized structure bridging the inner and outer segments, facilitating protein and vesicle trafficking critical for photoreceptor maintenance and visual function [3,4]. With advancements in molecular biology, eight genes—*NPHP1*, *NPHP3*, *NPHP4*, *IQCB1*, *CEP290*, *SDCCAG8*, *WDR19*, and *TRAF3IP1*—have been identified as pathogenic for SLSN, and their individual functions and overall roles in the primary cilium are gradually being understood. Furthermore, the genotype–phenotype correlation introduces further complexity to the concept of ciliopathies, driving ongoing genetic investigations. Based on clinical manifestations and molecular evidence, an increasing number of studies have concentrated on improving the prognosis of SLSN patients, particularly regarding their ocular phenotypes. Therefore, this review aims to examine the clinical characteristics and pathogenetic mechanisms of SLSN, explore the genotypic–phenotypic correlation of SLSN to improve early diagnosis, and summarize current advancements in treatment to inform future research directions.

## 2. Clinical Features of SLSN

### 2.1. Retina

The most common ocular manifestations of SLSN are Leber’s congenital amaurosis (LCA) and retinitis pigmentosa, both of which are characterized by progressive retinal degeneration that can ultimately result in complete vision loss [5]. Retinal degeneration can be divided into two clinical subgroups [6]. Early-onset SLSN typically manifests as LCA, exhibiting nystagmus, amaurotic pupils, and/or blindness in children before 2 years of age. A full-field electroretinogram often reveals markedly reduced or absent responses. The fundoscopic examination may appear normal in the early stages but progressively reveals abnormalities such as optic disc pallor, vascular attenuation, and peripheral pigmentary retinopathy over time [5,7,8]. In contrast, delayed-onset SLSN typically presents initially with night blindness and progressively advances to complete vision loss, similar to the clinical progression seen in retinitis pigmentosa. In these patients, electroretinography shows reduced amplitudes of the a- and b-waves, along with prolonged implicit times in rod- and cone-isolated responses [9,10]. The fundoscopic examination may reveal intraretinal pigmentation, often described as bone-spicule deposits, reflecting the migration of retinal pigment epithelium (RPE) cells following photoreceptor degeneration [9]. It is essential to note that the ocular phenotypes associated with SLSN exhibit considerable heterogeneity. While certain hallmark features mentioned above are commonly observed, the individual clinical presentations and examination findings largely depend on the underlying gene mutation and its specific location [5,8].

### 2.2. Kidney

NPHP is a chronic tubulointerstitial nephropathy with progressive renal failure, which is a leading cause of end-stage renal disease (ESRD) in children and adolescents. Patients with NPHP may present with increased urine output, nocturia, excessive thirst, weakness, and severe fatigue. NPHP can be classified into three clinical subgroups: infantile, juvenile, and adult forms, with a median age of ESRD at 1, 13, and >15 years, respectively [11]. Generally, juvenile and adult forms share similar clinicopathological characteristics. Classic ultrasound findings include normal or reduced renal size, loss of corticomedullary differentiation, and corticomedullary cysts [12]. Renal histology reveals a characteristic triad of tubular atrophy, diffuse interstitial fibrosis, and tubular basement membrane anomalies [13]. Some patients have kidney tissue biopsies showing some glomeruli with complete sclerosis, while others demonstrate compensatory hypertrophy, a manifestation of moderate chronic kidney disease [14]. Notably, infantile NPHP differs from typical NPHP, as renal ultrasound imaging may reveal kidney enlargement [15].

### 2.3. Others

Although SLSN is a ciliopathy characterized by retinal and renal dysplasia, it is widely pleiotropic, and clinicians should not overlook other accompanying symptoms. For example, developmental delay, hypertension, and premature ovarian failure are common in patients with SLSN, while abnormal bone density, ataxia, cone-shaped epiphyses, and congenital liver fibrosis occur occasionally [16]. In addition, other symptoms may vary across different patients. Su Ann Tay’s case report presents the first reported case of an SLSN patient with concomitant intracranial hypertension [17]. The heterogeneity of clinical symptoms may reflect the significant role of the primary cilium during individual development.

Currently, there is no cure for SLSN, but the presence of characteristic clinical complications, such as retinal degeneration and renal dysfunction, still enables clinicians to provide timely symptomatic and supportive interventions to improve patients’ quality of life.

## 3. Primary Cilium

### 3.1. General Structure and Function

The primary cilium is a microtubule-based, immotile organelle that protrudes as a single unit from the basal body, which is derived from the centrosomal mother centriole in most human cell types. Key structural components of the primary cilium consist of an axoneme, basal body, and transition zone (TZ).

**Figure 1:**
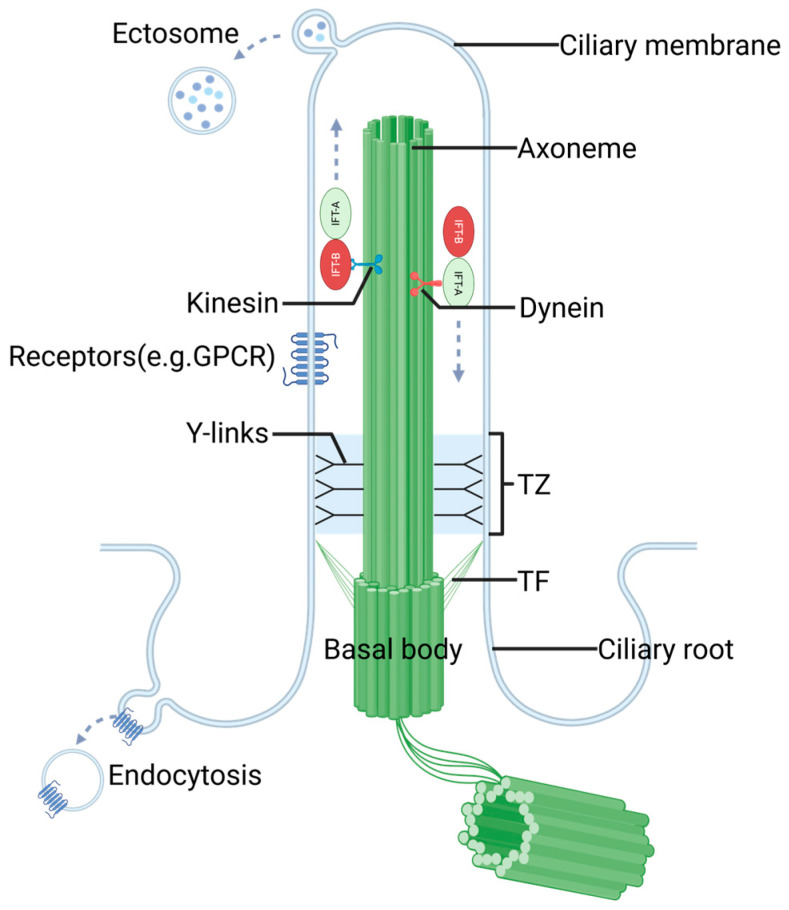
The schematic diagram of primary cilium structure and function. Abbreviations: TZ, transition zone; TF, transition fibers.

The axoneme of a primary cilium is composed of nine outer doublet microtubules, which are enveloped by the ciliary membrane. The ciliary membrane is contiguous with the plasma membrane and is enriched with specific signaling receptors and ion channels [18]. This structural specialization allows the cilium to detect changes in the extracellular environment and transmit signaling information to the cell, thereby regulating a wide range of cellular, developmental, and physiological processes [19]. Anchored to the ciliary root, the basal body is a modified centriole containing a ring of triplet microtubules and specialized structures named transition fibers at its distal end [20,21,22], which serve as an interaction platform and regulate critical aspects of the ciliary biogenesis and function [23,24,25]. Located between the basal body and the axoneme, the TZ is a crucial component of the primary cilium, containing specialized gating structures such as Y-links. Together with the transition fibers of the basal body, the TZ serves as a gating and docking station for proteins entering and exiting the primary cilium [26]. In addition to gating by the TZ and basal body transition fibers, the composition and function of the cilium are also regulated by active transport mechanisms (Figure 1).

Crucially, numerous genes mutated in ciliopathies, such as Joubert syndrome (JS), Meckel–Gruber syndrome (MKS), and NPHP encode protein components of the TZ or basal body transition fibers. This highlights the physiological significance of these structures [27].

### 3.2. Primary Cilia in the Eye

The capacity to generate cilia is present in nearly every human cell type, but not all cell types within a specific tissue or organ will produce cilia. Moreover, cilia formed in different cells may exhibit specificity, with their ciliary protein composition varying according to cell type and physiological state. While current research mainly focuses on cilia in photoreceptor cells, there are numerous other types of ciliated cells in the vertebrate eyes, encompassing the cornea, ciliary body, iris, lens, trabecular meshwork, and the RPE [28]. Although the biological role of these primary cilia remains incompletely understood, growing evidence suggests that cilia contribute to organogenetic tissue repair and vision [29,30,31].

### 3.3. Primary Cilium in Retinal Photoreceptors

**Figure 2:**
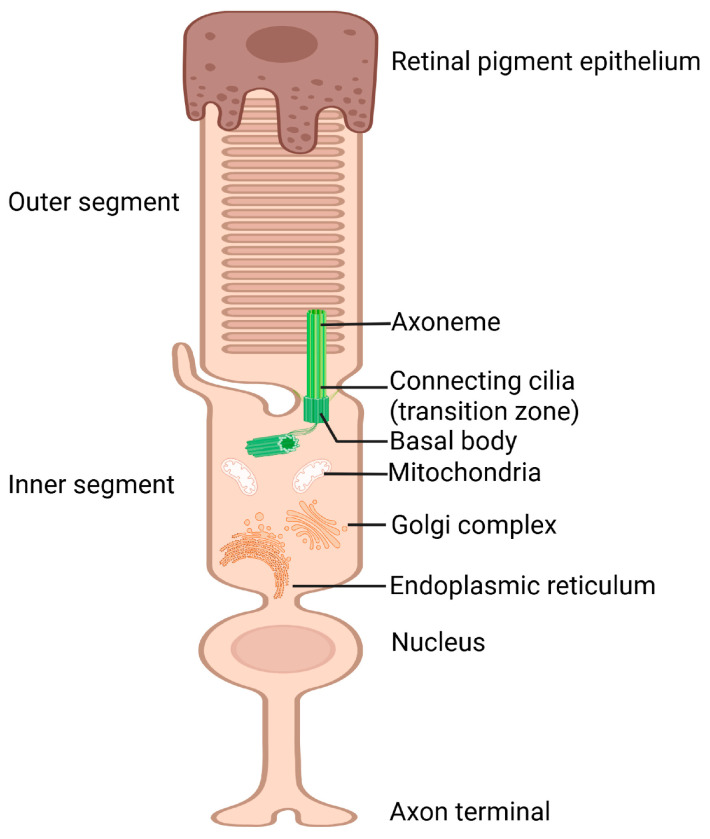
Schematic representation of the structure and function of primary cilium in photoreceptors.

Photoreceptors are specialized neurons in the retina that detect light and convert it into electrical signals. They have four distinct compartments: the outer segment (OS), which contains the light-sensitive molecules called rhodopsin in rods and opsin in cones, the inner segment (IS), which houses the metabolic machinery, the nucleus, which contains the genetic material, and a short axon that extends to second-order neurons [32]. To facilitate efficient phototransduction, primary cilia in the OS are specialized into photosensitive cilia. Both cone and rod photoreceptor outer segments contain hundreds of membrane discs that surround the axoneme and form coin-like stacks [33]. The IS houses mitochondria, rough endoplasmic reticulum, the Golgi apparatus, and microtubules, all of which play crucial roles in normal cellular functions [34]. The axoneme within the OS is securely anchored to the basal body located at the apex of the IS by a specialized TZ known as the connecting cilium, which is the sole direct link between the IS and OS of photoreceptors, facilitating the exchange of visual signaling molecules between the two compartments [3]. The outer segment’s apical surface is encased by the RPE (Figure 2). Within this environment, the apical membrane discs of the OS undergo both phagocytosis by the RPE and regeneration from the base. Furthermore, the RPE plays a crucial role in recycling retinol after phototransduction. PR cells exhibit a high degree of specialization and function in coordinated synchrony within the phototransduction cascade. As a result, any alterations, whether direct or indirect, caused by dysfunctional PRs that compromise the structural or functional integrity of this system can lead to progressive retinal degeneration. These changes may arise from variations in proteins found in the OS, IS, cilia, synapses, and mitochondria [35].

## 4. Pathogenesis Mechanisms

### 4.1. The SLSN Genes

SLSN displays both clinical and genetic heterogeneity. Since Senior and Loken first described the features of SLSN with nephronophthisis and Leber congenital amaurosis in 1961 [36,37], the Online Mendelian Inheritance in Man (OMIM) database has cataloged eight types of SLSN mutations in specific genes [38]. Table 1 provides a summary of the genes implicated in SLSN, along with their chromosomal locations and the corresponding encoded proteins [39,40].

#### 4.1.1. NPHP1

*NPHP1*, mapped to chromosome 3q12, encodes a 4.5-kb transcript and an 83-kDa protein, and was the first SLSN gene to be localized [65]. It encodes NPHP1, a cytoplasmic protein associated with cell–cell junctions, focal adhesions, and cilia [66]. Indeed, *NPHP1* plays a crucial role in organizing epithelial cells, as demonstrated by the disruption of apicobasal polarity upon *NPHP1* knockdown in Madin–Darby canine kidney cells and its interaction with BCAR1 in IMCD3 cells [67,68]. Besides its roles in adhesion and epithelial morphogenesis, the *NPHP1* product primarily regulates the transport of proteins bound to the OS/primary cilium, as well as overseeing a specific subset of IFT particles and their associated cargos destined for the OS, which is required for retinal development [69]. However, the precise molecular function of the NPHP1 protein is largely unknown, and the molecular defects underlying the pathogenesis of the disease remain unclear. The primary structure of NPHP1 supports its role as an adaptor protein, featuring a Src homology 3 (SH3) domain surrounded by two E-rich domains. These two domains interact with Crk-associated substrates and appear to play roles in regulating cell division and mediating cell–cell and cell–matrix adhesion signaling [65].

#### 4.1.2. NPHP3

The genomic region of the *NPHP3* gene, extending to 6.5 kb, which encodes a 1330-amino acid protein, has been proven to be expressed in the retina and contributes to the SLSN development [70]. This gene encodes a protein (NPHP3) that contains an N-terminal coiled–coil domain, a tubulin–tyrosine ligase domain, and a tetratricopeptide repeat domain [70]. Some studies have shown that the N-terminal myristoylation of the NPHP3 mediates cilia targeting [71,72]. Furthermore, Bergmann et al. demonstrated that the NPHP3 protein is essential for proper ciliary development and function. It inhibits Disheveled-1-induced canonical Wnt-signaling activity and is involved in controlling non-canonical Wnt signaling, which regulates planar cell polarity. Additionally, it acts as a molecular switch between various Wnt-signaling pathways [46].

#### 4.1.3. NPHP4

Mutations in the *NPHP4* gene, located on chromosome 1p36 and encoding a 1250-amino acid protein called NPHP4, can also cause SLSN [73]. NPHP4 interacts with retinitis GTPase regulator (RPGR) and RPGR-interacting protein 1 (RPGRIP1), potentially establishing a connection to the development of retinal degeneration in patients with an *NPHP4* mutation. Studies have shown that mutations in *NPHP4* disrupt the interaction and cause NPHP or LCA [74,75]. Interestingly, as an important ciliary protein, it does not appear to be strictly required for ciliogenesis in human cells but is essential for building functional cilia and involved in the organization of the subapical actin network in multiciliated *Xenopus laevis* epithelial cells [68,76]. In addition to its role in the visual cycle, the NPHP4 protein plays a crucial role in signal transduction. The conserved Hippo-signaling pathway regulates organ size in mammals and has an essential role in tumor suppression and the control of cell proliferation. Moreover, it functions as a negative regulator of the Hippo pathway by associating with LATS1 (large tumor suppressor 1), modifying LATS1-dependent phosphorylation, and leading to derepression of these protooncogenic transcriptional regulators [77]. In collaboration with NPHP2, the NPHP4 protein may downregulate the canonical Wnt pathway and promote the Wnt–PCP pathway by regulating the expression and subcellular localization of disheveled proteins. In addition, it stabilizes JADE1 protein levels and promotes its translocation to the nucleus, leading to cooperative inhibition of canonical Wnt signaling [78,79].

#### 4.1.4. IQCB1/NPHP5

*IQCB1*, also called *NPHP5*, spans 6.6 kb on human chromosome 3, consists of 15 exons, and encodes a 69 kD protein called nephrocystin-5/IQ calmodulin-binding motif-containing protein 1(IQCB1/NPHP5). The encoded protein possesses a central coiled-coil region and two calmodulin-binding IQ domains. It localizes to the primary cilia of renal epithelial cells and the connecting cilium of photoreceptor cells [2]. Especially, unlike patients with *NPHP1-4* mutation who have a 10–33% chance of developing retinal degeneration, 100% of patients with *IQCB1* mutation are thought to develop SLSN [80]. Moreover, in contrast to other SLSN genes, which may affect multiple organs, *NPHP5* mutations only associate with the retinal–renal phenotype and are the most frequent cause of SLSN, making it the classic SLSN gene [80,81]. The severity and high prevalence of eye complications in patients with *IQCB1* mutations may be attributed to the critical role of the encoded protein in ciliary function. Apart from IQ domains and CC domains, NPHP5 has a CEP290/NPHP6-binding site and Bardet–Biedl syndrome (BBS) interaction sites [81]. Through its interaction with CEP290, the IQCB1 protein is involved in ciliogenesis and functions in early-stage cilia formation [82]. Proper assembly of BBSome plays a crucial role in ciliary transport and formation in both photoreceptor cilia and non-photoreceptor primary cilia [83]. The IQCB1 protein regulates the BBSome complex integrity, specifically ensuring the presence of BBS2 and BBS5 within the complex and their ciliary targeting of selected BBSome cargos [83]. Knockdown of *IQCB1* in zebrafish can ultimately cause the mislocalization of opsins and rhodopsin to the IS, as well as OS dysplasia in mice, suggesting its role in the specific transport of proteins to the OS and IS development [81,84].

#### 4.1.5. CEP290/NPHP6

*CEP290*, also known as *NPHP6*, is situated on chromosome 12q21.32 and encodes a 290 kDa centrosomal protein (CEP290/NPHP6) [85]. This protein is characterized by 13 putative coiled-coil domains, a region exhibiting homology to SMC chromosome segregation ATPases, six KID motifs, three tropomyosin homology domains, and an ATP/GTP binding site motif A. It specifically localizes to the basal bodies of photoreceptors, facilitating the linage of the cilium and significantly contributing to its formation, stability, and transport function [5]. Due to its important role in ciliogenesis, *CEP290* is a major focus among the pathogenic genes associated with SLSN. It is required for both early and late steps in cilia formation through its interaction with the IQCB1 protein [86]. Furthermore, CEP290 plays a significant role in the early stages of ciliogenesis, involving the disappearance of centriolar satellites and the transition of primary ciliary vesicles to capped ciliary vesicles where it is essential for the centrosomal recruitment of RAB8A and the targeting of centriole satellite proteins, such as PCM1, to the centrosome [87,88]. In cooperation with IQCB1, the IQCB1 protein also contributes to the assembly of BBS complexes [89].

#### 4.1.6. SDCCAG8/SLSN7/NPHP10

*SDCCAG8*, also known as *SLSN7* or *NPHP10*, produces a 3.3 kb transcript that encodes an 88 kDa protein called SDCCAG8. This protein comprises an N-terminal globular domain, a nuclear localization signal, and eight potential coiled-coil domains [90]. The carboxyl-terminal region of SDCCAG8 contains a crucial functional module essential for cilia formation, organ development, and homeostasis [91]. Previous studies have focused on its role in BBS syndrome [57,92], but its significance in SLSN should not be ignored. During ciliogenesis, it interacts with RABEP2 and mediates its centrosomal localization. Furthermore, SDCCAG8 is required for the proper activation of the Hedgehog signaling pathway, which depends on the presence of intact primary cilia [93].

#### 4.1.7. WDR19

*WDR19* contains 37 exons and encodes three different types of domains in the IFT144 protein: six WD40 domains, which are short structural motifs of 40 amino acids ending in the tryptophan–aspartic acid dipeptide, two transmembrane domains, and six tetratricopeptide repeat domains. The common function of all WD40-containing proteins is the coordination of the assembly of multi-protein complexes, while tetratricopeptide repeat domains mediate protein–protein interactions within these complexes [94]. The IFT144 protein is a component of the IFT-A complex, which plays a crucial role in the retrograde transport of cargo within the cilium [60]. This protein is primarily expressed in the retina, particularly in photoreceptors, but also in various other tissues. In this context, WDR19 acts as a component of the IFT-A complex, which is essential for retrograde ciliary transport and the entry of G protein-coupled receptors into the cilium. This involvement in retrograde transport is critical for maintaining the cilia function and assembly [95].

#### 4.1.8. TRAF3IP1

The *TRAF3IP1* gene encodes the homolog of the intraflagellar transport protein IFT54, a subunit of the IFT-B complex that facilitates anterograde transport within cilia [62]. This 625-amino acid protein contains a KKE motif and a C-terminal coiled-coil domain [96]. Mutations in the N-terminal region of TRAF3IP1 impair its ability to bind to MAP4 [62]. Notably, MAP4 is also localized within primary cilia, where it functions as a negative regulator of ciliary length, counteracting the activity of septins [97].

### 4.2. The SLSN Interactome

The SLSN interactome encompasses several ciliary protein modules that converge at TZ, a critical gatekeeper for ciliary compartmentalization and signaling. Among these, the NPHP1-4-8 and NPHP5-6 complexes are two canonical modules essential for TZ integrity and are widely expressed in cilia-dependent tissues, such as retinal photoreceptors, kidney tubular epithelial cells, and cerebral cortical neurons. Dysfunction of these modules results in a spectrum of ciliopathies, including SLSN. Elucidating their molecular mechanisms will enhance our understanding of the retinal pathogenesis of SLSN and provide a basis for the development of targeted therapies.

#### 4.2.1. NPHP1-4-8 Complex

Functioning as a classical SLSN-related module, the NPHP1, NPHP4, and NPHP8 protein share many common characteristics:

- Containing multiple C2 domains in their core regions. These C2 domains play a central role in the membrane association at the ciliary transitional zone and serve as the protein–protein interaction modules connecting different transition zone proteins [98,99].
- Co-dependent localization. For example, the loss of NPHP-4 leads to the delocalization of NPHP-1 [100], and a similar situation occurs between NPHP8 and NPHP1 [101,102], as well as between NPHP4 and NPHP8 [51].
- Elegant connections with microtubules. NPHP1, NPHP4, and NPHP8 interact with β-, α-, and γ-tubulin, respectively, which happen to be the three main components of microtubules [103,104].

These characteristics enable the NPHP1-4-8 complex to locate proximally to the microtubule sides in the TZ [27], where it participates in both its composition and function. Since the connecting cilium (CC) in photoreceptors is the equivalent of the TZ, which is the only direct link between the ISs and Oss [105], alterations in NPHP1-4-8 complex may be a core mechanism underlying the ocular phenotypes of SLSN. Structurally, defects in the NPHP1-4-8 complex can damage the Y-link structure at the TZ [41], thus impairing its gating function [106]. Moreover, the NPHP1-4-8 complex seems to regulate the cargo transport in the CC, since the defects in each protein of the complex can cause the sluggish movement of the IFT [27,41,69,107], which may indicate that the complex mediates the interaction between TZ and IFT particles [41,69,108]. The RPGR provides evidence for this explanation. It extensively interacts with the NPHP1-4-8 complex and acts as a guanine nucleotide exchange factor in modulating the rhodopsin trafficking in photoreceptors [109,110,111]. The defects of NPHP1-4-8 can impair its interactions with RPGR, leading to the mislocalization of rhodopsin [75,112] and ultimately resulting in the dysfunction of photoreceptors, which is the underlying cause of ocular phenotypes in SLSN.

Notably, NPHP8 seems to act as a central scaffold at the TZ, functionally linking the NPHP and other modules [101,102,106]. Hence, the mutations in *NPHP8* tend to cause more severe clinical phenotypes, making it a secondary pathogenic gene in SLSN. Rather, the mutations in *NPHP1* and *NPHP4* cause only mild defects in ciliogenesis and lead to less severe clinical phenotypes [27,41]. This theory is supported by a large-sample clinical study [113].

Moreover, NPHP1, NPHP4, and NPHP8 have also been found to accumulate at cell–cell contacts and participate in the process of cell polarity, particularly during the development of polarized epithelial monolayers [67,68], which may represent an additional mechanism independent of ciliopathies. However, most research only focuses on the NPHP1-4 complex, demonstrating its ability to regulate epithelial morphogenesis by interacting with polarity proteins such as the PALS1/PATJ complex and p130Cas [67,114]. Fortunately, recent studies have shown that NPHP8 is required for stabilizing epidermal adhesion and participating in the cell polarity process [115,116]. This may indicate the combination of NPHP1-4-8 complex outside the primary cilium.

#### 4.2.2. NPHP5-6 Complex

Functional studies have demonstrated that NPHP5 interacts strongly with NPHP6, binding to its N-terminal region via its C-terminal domain [82,117]. In both mammalian cells and IMCD3 cells, they have been proven to be located in centrosomes. Additionally, studies have concluded that NPHP6 binds NPHP5 and facilitates its localization to centrosomes [68]. However, subsequent research using a large panel of mutants revealed that the NPHP6-binding domain in NPHP5 is distinct from its localization domain [82]. These findings show that while some mutants disrupting the NPHP6-binding domain impair cilia formation, they do not directly contribute to mislocalization [82]. While earlier studies proposed that NPHP5 forms a complex with NPHP6 and calmodulin, recent research demonstrates that a drug named Eupatilin can disrupt the CaM–NPHP5 interaction, promoting NPHP5 centrosomal localization in *NPHP6*-null cells [118]. This suggests that while NPHP6 influences NPHP5 localization, the mechanism is indirect.

NPHP5 and 6 form a complex to play a role in ciliogenesis and protein trafficking [82,89]. For example, the complex can regulate BBSome integrity and ciliary trafficking. Both of them can independently bind to BBSome, and the deletion of *NPHP5* leads to a loss of BBS2 and BBS5, while the deletion of *NPHP6* causes the dissociation of BBS8 from BBSome [89,119]. Moreover, the complex functions in ciliogenesis by copurifying with ciliogenesis-required proteins such as Sec3, Sec8, and Sec10 [120].

Findings from recent pharmacological rescue research indicate that NPHP5 and NPHP6 coordinately influence a common pathway converging on ciliogenesis and protein trafficking. Drugs such as cytochalasin D, which rescue the *NPHP5*-deletion phenotype, exhibit a similar effect on the *NPHP6*-deficient phenotype [82]. However, there is recent research showing that Eupatilin can only have a rescuing effect by restoring NPHP5 levels in *NPHP6*-null cells [89,118]. BBSome integrity can be rescued by *NPHP5* overexpression in *NPHP6*-null cells [121]. Moreover, the ciliary gating can be controlled by Rpgrip1l via maintaining appropriate levels of CEP290 at the TZ [122]. As a result, there is still a significant difference between NPHP5 and NPHP6, which is unclear. Figuring out the proteins “downstream” and their binding mechanisms with the complex of NPHP5 and NPHP6 may be a thoughtful way for dissecting their biological roles and functions [82].

## 5. Genetic Basis

### 5.1. Shared Phenotypes Among Ciliopathies

**Figure 3:**
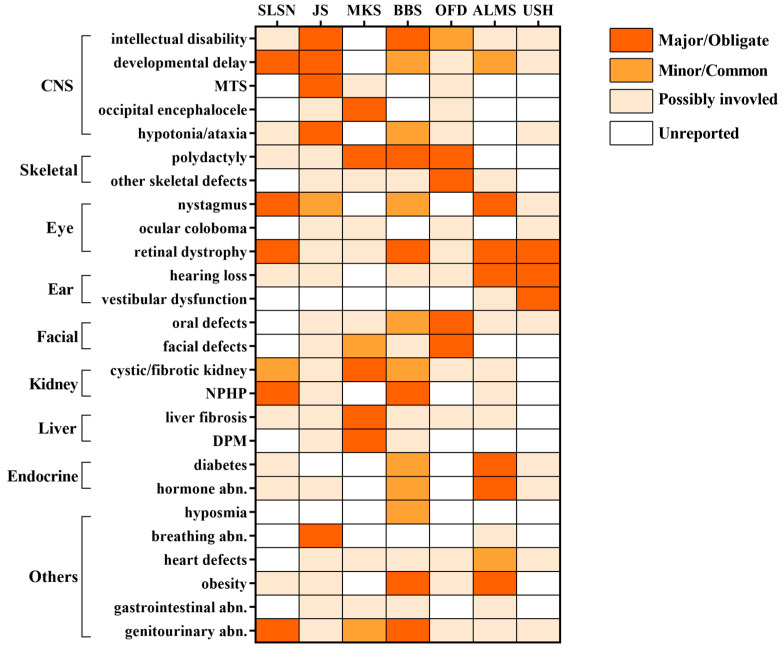
Clinical overlap and distinctions between SLSN and other ciliopathies. The typicality and frequency of features in different syndromes are reflected through the gradation of three colors (the darkest means major/obligate features, the medium means minor/common features, and the lightest means possibly involved features), while some features not yet reported are left blank. Abbreviations: SLSN: Senior–Loken syndrome; JS: Joubert syndrome: MKS: Meckel syndrome; BBS: Bardet-Biedl syndrome; OFD: Oral-facial-digital syndrome; ALMS: Alström syndrome; USH: Usher syndrome; CNS: central nervous system; MTS: molar tooth sign; NPHP: nephronophthisis; DPM: ductal plate malformation. abn.: abnormalities. Reference: SLSN: [6,35,123,124,125]; JS: [35,128,129,130,131,132,133]; MKS: [128,129,132,134]; BBS: [35,128,129,135,136]; OFD: [137,138,139]; ALMS: [140,141,142,143,144]; USH: [145,146,147]; Other information comes from the Human Phenotype Ontology (HPO) database.

Although the clinical phenotypes of SLSN are mainly concentrated in the eyes and kidneys, it is clinically heterogeneous with variable multiorgan involvement, which shares extensive overlap with other ciliopathies, such as Joubert syndrome, Meckel syndrome, Bardet–Biedl syndrome, oral–facial–digital syndrome, Alström Syndrome, and Usher syndrome. Additional shared phenotypes include CNS (intellectual disability, developmental delay, ataxia), skeletal(polydactyly), ear (hearing loss), liver (liver fibrosis), endocrine (diabetes, hyperinsulinemia), and obesity [6,35,123,124,125,126,127] (Figure 3). 

The remarkable phenotypic overlap among these ciliopathies is rooted in the shared molecular biology foundation. Firstly, the primary cilium exists in the development process of most human cells, which is highly conserved and essential for regulating signaling pathways during development and adult homeostasis, so the defects of the primary cilium can cause multiple organic dysfunctions [148,149]. Additionally, ciliopathy proteins function as complexes and interact modularly in the biogenesis of the primary cilium [6,150,151,152], so mutations in genes encoding SLSN proteins can disrupt their interaction with other ciliopathy protein modules, eventually causing similar multi-organ phenotypes in analogy with other ciliopathies.

Recently, some research has proposed a new perspective that the phenotypic spectrum in these ciliopathies is more of a continuum rather than distinct syndromes, emphasizing the importance of considering the broader context, instead of sticking to a specific syndromic diagnosis based on the presence of certain phenotypes [133,153].

### 5.2. Genotype–Phenotype Correlations

In some ciliopathies caused by mutations in certain genes, significant gene–phenotype correlations have been observed. For example, mutations in *CEP290* are always associated with retinal dystrophy, while mutations in *WDR19* are often linked to skeletal anomalies [58,154,155,156,157]. However, considering its extensive phenotypic overlap with other ciliopathies and remarkable clinical heterogeneity, it is obvious that SLSN, like most ciliopathies, extends beyond the simple one-gene-to-one-phenotype relationship. To explain these complex correlations, different genetic mechanisms have been proposed, some of which have been identified in SLSN.

#### 5.2.1. Locus Heterogeneity

Mutations in different genes can cause similar phenotypes, and this potential is amplified in ciliopathies due to the modular expression of ciliopathy genes and related proteins [158]. However, similar phenotypes do not necessarily indicate similar severity, and the latter is determined by specific genes [159]. For example, NPHP1, NPHP4, and NPHP8 are all found to localize to the TZ as a complex [160], and mutations in genes encoding any of them can lead to NPHP and potential extrarenal organ manifestations, but variants of *NPHP1* seem to link with the lowest extrarenal performance rate and the mildest disease phenotype [113].

#### 5.2.2. Allelic Heterogeneity

Different mutations in the same gene can cause distinct phenotypes. If two truncating mutations act during development, they may affect morphogenesis and cause a severe, early-onset phenotype, characterized by organ dysplasia or malformation; whereas two missense mutations act during tissue maintenance and repair in adult tissue, they may proceed slowly and finally cause organ degeneration [6]. This probably explains why individuals with SLSN have significant differences in the onset of the eye and kidney syndromes [64]. The correlations between hypomorphic mutations and mild phenotypes have been identified in several studies [153,161,162]. One striking example is a patient with compound heterozygous variants *p.D1781E* and *p.T412M* in *CEP290*, who only suffered mild RP and retained good vision at the age of 55 years [162]. However, the association between the nature of mutations and phenotypes is still challenged in some cases. For example, patients with two truncating mutations in *CEP290* suffer from diseases ranging from mild SLSN, JS to lethal MKS [163,164,165]. This may imply that the mutation domain within the protein is of the same importance [113,166], although there may exist other mechanisms such as genetic modifiers and oligogenic inheritance.

#### 5.2.3. Genetic Modifiers and Epistatic Effects

Genetic modifiers and epistatic effects describe the effects of one gene/allele on the phenotypic outcome of another gene/allele [167], which is another explanation for the genotype–phenotype complexity. A case in point is the allele *A229T* of *RPGRIP1L*, which has been found to be enriched in SLSN patients, can intensify the development of retinal degeneration by decreasing the stability of RPGRIP1L-RPGR complexity in the context of mutations in specific ciliopathy proteins [168]. Also, mutations in *AHI1* (especially its variant *p.R830W*) seem to play a similar role in SLSN [169]. With the development and application of next-generation sequencing, more modifiers and epistatic effects have been reported in ciliopathies [53,170,171], contributing to a better understanding of the impact of genetic background. However, although genetic modifiers are collectively common, they may be individually rare and heterogeneous. Hence, they are unlikely to be discovered when studied in isolation [172], which provides research directions for some multicenter, large-sample cohort studies.

#### 5.2.4. Oligogenic Inheritance

Different from genetic modifiers, oligogenic inheritance requires the co-inheritance of mutations in two non-alleles on separate genes, which together are both necessary and sufficient to cause the phenotype [159,167,173]. “True oligogenic inheritance” was confirmed in BBS [174,175], and some evidence was found in NPHP [176]. However, in most studies, it is difficult to distinguish whether a phenotype derives from genetic modifiers or oligogenic inheritance, and sometimes the borderline between them is blurred [170,177], considering the unpredictable polymorphic nature of the human genome and limited genetic analyses [172,173]. In fact, both mechanisms support the hypothesis of the mutational load, in which the expressivity and penetrance of ciliopathies in an individual are determined by their own genetic background, with potential additional variants in other ciliary genes [178]. The co-occurrence of distinct ciliopathy phenotypes within a single family provides an elegant case [176,179].

Although it seems that ciliopathies mostly obey classic Mendelian inheritance [180], the potential effects of variants in genetic backgrounds should never be underestimated [181]. In this context, NGS may be an effective method for improving diagnostic accuracy, identifying pathogenic mechanisms, and eventually providing guidance for clinical therapy.

## 6. Treatments

### 6.1. Gene Therapy

At present, the main methods of gene therapy for SLSN can be divided into gene substitution and gene editing. For gene substitution, the most widely used and efficient delivery vector is the recombinant adeno-associated virus (AAV) [182,183]. AAV-mediated *NPHP5* gene augmentation has been confirmed to be capable of achieving therapeutic effects in a dog ciliopathy model of Leber congenital amaurosis, which provides a possible way for the treatment of SLSN in humans [184]. Yet, it cannot deliver genes of large sizes owing to its limited packaging capacity. Consequently, it is an alternative choice to deliver only a partial gene encoding a specific functional domain impacted by mutation(s) rather than a full-length gene. Research has also proved that a *CEP290* fragment representing a part of the most important SLSN-causing genes can reconstitute *CEP290* function and result in cone preservation and delayed rod death [185]. This finding suggests that the approach of delivering partial gene fragments holds implementation potential. However, genetic heterogeneity precludes gene therapy based on the delivery of the same gene to all SLSN patients. Hence, any future gene therapy would need to be personalized based on which gene is mutated in each patient [186,187,188].

Moreover, SLSN caused by mutations in *CEP290* can be potentially treated by gene editing using CRISPR/Cas9, which offers a viable solution to the limited packaging capacity of AAV [189]. CRISPR/Cas9 stands as the most representative method in current gene-editing therapies. Its high-precision editing has transformed ocular gene-therapy paradigms, allowing for the development of disease models and targeted correction of pathogenic mutations to drive therapeutic progress [187]. EDIT-101 is a CRISPR/Cas9-based gene-editing therapy designed for *CEP290*-associated inherited retinal diseases. Preclinical research confirmed EDIT-101’s mechanistic feasibility in vitro, demonstrating targeted nuclease activity in human cells and retinal tissues, while in vivo models including mice and non-human primates showed effective, long-lasting gene editing and therapeutic efficacy [188]. Building on these preclinical milestones, a Phase 1–2 clinical trial subsequently evaluated EDIT-101 in 14 patients with *CEP290*-related IRDs, revealing favorable safety profiles and meaningful improvements in visual function [186]. Significantly, these outcomes have established a robust foundation for advancing subsequent CRISPR-based translational research in this field.

In addition, RNA-based therapies, such as RNA interference and antisense oligonucleotides, can be considered to overcome the limitations of gene delivery, which have been shown to be more economical and efficient than DNA-based therapies [190,191,192]. Specifically, for SLSN, antisense oligonucleotide technology has already been tested in a humanized mouse model of *CEP290*-LCA. The results demonstrated that it can reduce the expression of erroneous mutations, thereby increasing cilia length and improving their functionality [193,194].

### 6.2. Drug Discovery

To date, SLSN has been linked to mutations in multiple disease-causing genes, with novel pathogenic variants continuously being identified [15]. Consequently, as previously discussed, tailoring gene therapy to individual mutations remains challenging and less feasible due to genetic heterogeneity. SLSN is a condition characterized by retinitis pigmentosa and NPHP [64,134,160], making symptom-targeted therapies a promising alternative approach.

For the purpose of slowing retinal degeneration and maintaining patients’ vision, neuroprotective agents are an excellent choice, which includes neurotrophic factors, anti-apoptotic agents, and antioxidants [195]. Supplements of antioxidants such as docosahexaenoic acid and vitamin A have shown the potential in preventing photoreceptor apoptosis [196,197,198]. Moreover, the ciliary neurotrophic factor, a type of neurotrophic factor, is one of the most effective cytokines in slowing retinal degeneration. It prevents protein degradation to protect the retinal cells from death [199]. And tauroursodeoxycholic acid has also shown potential to be applied in the treatment of retinitis pigmentosa as an anti-apoptotic agent [200]. In addition to therapies targeting ocular symptoms, antibiotic administration can be used to reduce inflammation and ease symptoms.

Beyond drugs focusing on the clinical manifestations of SLSN, pharmacological modulators that act on ciliary-signaling pathways and enhance ciliary stability offer a complementary approach as well, particularly to address the limitations of gene therapy. Treatment with the small molecules eupatilin and fasudil reduces the accumulation of CEP290 and IFT88 along cilia, improving rhodopsin trafficking to photoreceptor OSs and reducing its mislocalization in the outer nuclear layer [201]. These effects suggest that both drugs can target ciliary protein homeostasis, thereby holding promise for treating SLSN and other ciliopathies driven by ciliary dysfunction [118,202,203]. Previous research has reported that the microtubule-targeting agent MI-181 (mitotic inhibitor-181) is a potent modulator of cilia length and biogenesis [204]. Although its direct efficacy in SLSN remains unproven, MI-181’s ability to restore ciliation at low concentrations without toxicity suggests the potential for treating ciliopathies.

### 6.3. Other Discoveries

Recently, optogenetics has shown the potential for the treatment of retinal diseases, involving the introduction of optic proteins into the degenerated retina to restore its function and confer photosensitivity to residual retinal cells [205]. Up to now, it has been proven to successfully restore vision in mice [206]. Thanks to the continuous developments, such as photosensitive proteins with near-infrared activation wavelengths, it may hold promise for treating SLSN, which is manifested as treatable retinal degeneration [207].

Moreover, when organ function is partly or completely lost, partial or complete organ replacement is a reasonable choice. In the case of retinal diseases, before the complete loss of rod and cone photoreceptors, cell transplantation offers a promising approach. In this method, normal cells are transplanted to replace functionally impaired cells [208]. For example, the results of implanting RPE monolayers differentiated from human embryonic stem cells into the sub-retinal space of patients with AMD have been shown to improve vision [209]. Stem cell therapy is an evolving technique, and further exploration is expected to lead to a promising prospect for SLSN treatment. In cases of severe photoreceptor loss or even complete degeneration, artificial retina implantation can be an alternative strategy. Although it can simulate the function of the natural human retinas, there still remain problems like cytotoxicity and the disruption of the blood–retinal barrier [210,211]. As a result, there is still a long way to go for the safety assurance and promotion of retinal transplantation.

As we already know, phenotypic heterogeneity in SLSN arises from mutations in different genes, which suggests that precision medicine targeting specific families and individuals will become the future direction of SLSN treatment development [64]. Complementing this approach, cilia-targeted therapies offer a promising strategy to address shared pathogenic mechanisms across different SLSN genotypes. These interventions broadly encompass three categories: (1) restoring normal ciliary function, as demonstrated by ectopic expression of arl3, arl13b, and unc119b to partially rescue photoreceptor OS abnormalities in *CEP290*-mutant zebrafish; (2) compensating for disrupted ciliary signaling, such as rebalancing aberrant Wnt/β–catenin pathways—implicated in renal cystogenesis in *CEP290/NPHP4* mutation models—via targeting GSK3β or the Wnt co-receptor LRP6; and (3) correcting or bypassing genetic defects underlying ciliary dysfunction, including the aforementioned gene substitution or editing strategies [186,212,213]. However, most of these approaches remain in preclinical stages or are primarily focused on renal phenotypes, with few clinical trials targeting ocular manifestations, highlighting their untapped potential for further translational development.

In conclusion, regardless of the treatment modality, standardized and precise medical treatment based on individual mutations and phenotypes should be conducted. At the same time, early diagnosis and regular testing are also indispensable parts of treatment.

## 7. Animal Models

Similar to the advances in molecular diagnosis and gene therapy, animal models have significantly contributed to the exploration and research on SLSN, especially to the research on mechanisms. For example, some researchers have created a knock-in mouse model carrying truncating mutations of the mouse *SDCCAG8* gene by using homology-directed recombination technology, which corresponds to SLSN-causing mutations in humans, respectively. Mouse embryonic fibroblasts from the knock-in mice exhibited impaired biogenesis and structural defects in cilia, which ultimately caused major phototransduction protein mislocalization outside the OS [214]. Moreover, a germline *NPHP5*-knockout mouse model was generated by placing a *β-Geo* gene trap in intron 4, removing all known functional domains. This model revealed that NPHP5/IQCB1 is necessary for mouse photoreceptor OS formation in mice [81]. Additionally, the function of *CEP290* has been explored by using a mouse model of LCA with a disrupted microtubule-binding domain, which caused significant deficits in cilia formation and retinal degeneration [215]. In addition to the exploration of pathogenic mechanisms for commonly studied genes, there are mouse models for other SLSN-associated genes, revealing differences in gene expression that likely contribute to phenotypic diversity and offering new insights into the disease’s pathogenesis and individual variability [46,68,107,216,217].

Constantly, researchers are developing new mouse models, such as a mouse model deficient in *TMEM218*, whose major observed phenotypes are similar to those observed in SLSN. As a result, this mouse model may provide a promising prospect for elucidating the pathogenesis of diseases like SLSN, as well as exploiting new therapeutic strategies [218].

Actually, abundant research on pathological mechanisms aims at paving the way for the development of treatments, and currently, some studies using mouse models are directly exploring therapeutic techniques. For instance, by generating the *Nphp5^–/–^; Nrl^–/–^* double-knockout mouse model, rat cones persistently express cone pigments in the ISs without obvious degeneration, which is extremely important for prolonging the duration of gene delivery and expression and provides hope for human SLSN gene therapy [219]. Similarly, the *CEP290* mouse model mentioned above provides a reasonable way for solving problems of the limited capacity of delivery carriers in gene therapy by delivering only the functional domain impacted by mutations [215].

Apart from the most researched mouse models, there are also other animal models used for SLSN relative exploration. Naturally occurring SLSN-causing mutations in genes such as *CEP290* and *ICQB1* have been identified in retinal degeneration cat models [220,221]. Zebrafish models with gene knockdown are also commonly used in the exploration of the pathogenic mechanism of SLSN [71,85]. Furthermore, a naturally occurring *NPHP5* mutation in dogs causes disease similar to SLSN in humans, and, thus, its gene therapy could simulate and predict the efficacy of human SLSN gene treatment [184].

## 8. Discussion

As one of the ciliopathies mainly characterized by retinal degeneration and glomerulonephritis, SLSN was first discovered in the 1960s. However, its pathogenic mechanism is not completely clear now. With respect to the ocular phenotype of SLSN, current research mainly focuses on the connecting cilia of photoreceptors, especially the NPHP1-4-8 complex and the NPHP5-6 complex. The destruction of these structures will lead to the disruption of the corresponding functions of CC, thus triggering retinal degeneration. However, certain details remain unclear. For example, why does the degeneration of NPHP5-6, unlike the NPHP1-4-8 complex, cause a 100% incidence of retinal degeneration [80]? Moreover, other mechanisms may be involved in photoreceptor degeneration. The latest research shows that RPE, an epithelial cell that maintains the physiological cycle of photoreceptors, also has many primary cilia during development, and the impact of RPE primary cilia degeneration may occur before retinal photoreceptor defects. The role of this degeneration in SLSN may be one of the directions for future research [222].

Interestingly, with the discovery and definition of more and more ciliopathies, it is clear that they share extensive phenotypic overlaps. These connections can be explained by the molecular mechanisms and genetic basis of these disorders. As previously discussed, there exist interactions and connections between the NPHP1-4-8 complex and the NPHP5-6 complex. In fact, as a part of the TZ, they also mediate interactions with MKS, JBS proteins, and BBSome complexes. Whether these proteins that cause ciliopathies all perform biological functions as a whole remains to be verified. The genetic mutation load found in large cohorts provides strong support for the hypothesis that the severity of an individual’s overall ciliopathy phenotype is related to the population of mutations in his or her ciliopathy-associated proteins [178].

The above phenomena increasingly support the idea that ciliopathies are essentially a unified continuum of phenotypes, which increasingly emphasizes the overall concept of ciliopathies and weakens the fragmented definition of ciliopathies by phenotype [131]. This should inspire clinicians to focus on molecular and genetic diagnosis, especially the judgment of mutation load, in order to provide individualized and precise treatment.

In fact, there is no complete and targeted fundamental solution for the treatment of fibrotic disorders, and most treatments are based on supportive treatment of relevant fibrotic symptoms, and the same applies to the treatment of SLSN. For SLSN, the current treatment still focuses on the treatment of the eyes, with dialysis transplantation being the main treatment for the kidneys. There are also drugs that have therapeutic effects on the kidneys, but their effectiveness in treating eye lesions in SLSN needs to be studied and explored [160]. In clinical research, the study of these drugs on the eyes and kidneys is often fragmented, lacking an integrated approach, which is undoubtedly detrimental to the treatment of SLSN [160]. In addition, when developing eye medications, attention should also be paid to whether these agents have nephrotoxicity, which is particularly important for the treatment of SLSN that may already lead to renal impairment.

Numerous new treatments have been developed for retinitis pigmentosa in terms of supportive ocular therapy, including optogenetic therapy. However, there have been no reports on its treatment for SLSN, highlighting the need for further experimental investigations to assess its effectiveness [206,207].

Gene therapy can solve the problem of symptomatic supportive therapy, especially for the delivery of certain gene fragments, making it a promising area of research. However, there are still significant challenges in the clinical application of gene therapy, such as personalized treatment for patients with multiple modified genes and mutations, which requires considerable progress before it can become a routine therapeutic option [192].

Moreover, current animal model research remains largely unsystematic. While numerous models exist, their characterization is often fragmented. These models typically focus on isolated phenotypes or pathways rather than comprehensive disease modeling, which complicates their translational utility. A critical limitation is the uncertainty regarding functional differences in orthologous proteins between humans and model organisms, casting doubt on the relevance of animal studies for understanding human SLSN pathogenesis and guiding therapeutic development.

## 9. Conclusions

As a ciliopathy, the ocular mechanisms and genetic features of SLSN have yet to be thoroughly elucidated through systematic exploration. Current research has predominantly concentrated on the primary cilium structure in retinitis pigmentosa. Alterations in SLSN-associated proteins disrupt the biogenesis and structural assembly of the primary cilium in these tissues, thereby impairing signaling and molecular trafficking. Due to the intricate protein interactions, SLSN gene mutations often exert widespread effects, with clinical manifestations that overlap considerably with other ciliopathies. Additionally, despite recent advances in gene therapy and optogenetics, major challenges remain in translating these approaches into effective clinical interventions for SLSN. A deeper understanding of its ocular pathophysiology and genotype–phenotype correlations is crucial to refining therapeutic strategies and ultimately improving patient outcomes.

## Figures and Tables

**Table 1:** Summary of the identified SLSN genes.

Gene	MIM Number	Chromosome Location	Protein	Protein Function	Localization in Cilium	Phenotype
*NPHP1*	266900, 607100	2q13	Nephrocystin-1	Cell-cell and cell-matrix signaling	Transition zone [41]	NPHP, SLSN, JS, BBS [42,43,44]
*NPHP3*	606995, 606995	3q22	Nephrocystin-3	Wnt signaling	Inversin compartments [45]	NPHP, SLSN, MKS, BBS, JS, MSS, COACH, SRTD [46,47,48,49]
*NPHP4*	606996, 607215	1p36.31	Nephrocystin-4	Wnt signaling	Basal body and transition zone [41]	NPHP, SLSN [50,51]
*IQCB1*	609254, 609237	3q13.33	IQ calmodulin-binding motif-containing protein-1	Ciliogenesis, regulation of BBSome complex integrity	Basal body [27]	LCA, NPHP, SLSN, JS [2,52,53]
*CEP290*	610189, 610142	12q21.32	Centrosomal protein of 290 kDa	Ciliogenesis, regulation of BBSome complex integrity	Transition zone [27]	LCA, NPHP, SLSN, JS, MKS, BBS [54,55]
*SDCCAG8*	613615, 613524	1q43-44	Serologically defined colon cancer antigen 8	Ciliogenesis, Hedgehog signaling	Basal body [56]	NPHP, SLSN, BBS [17,57]
*WDR19*	616307, 608151	4p14	WD repeat-containing protein 19	Ciliary transport, assembly	IFT-A complex [58]	NPHP, SLSN, ATD, Sensenbrenner syndrome, Caroli syndrome [58,59,60,61]
*TRAF3IP1*	616629, 607380	2q37.3	TRAF-3 interacting protein 1	Cilia import	IFT-B complex [62]	NPHP, SLSN, SRPS, ATD [63,64]

Abbreviations: LCA: Leber congenital amaurosis; SLSN: Senior–Loken syndrome; JS: Joubert syndrome: MKS: Meckel syndrome; MSS: Mainzer-Saldino syndrome; BBS: Bardet-Biedl syndrome; NPHP: nephronophthisis; SRTD: short-rib thoracic dysplasia; ATD: asphyxiating thoracic dystrophy; SRPS: short-rib polydactyly syndrome.

## Data Availability

No new data were created or analyzed in this study. Data sharing is not applicable to this article.

## Abbreviations

The following abbreviations are used in this manuscript:

SLSN	Senior–Loken syndrome
LCA	Leber’s congenital amaurosis
NPHP	Nephronophthisis
ESRD	end-stage renal disease
TZ	transition zone
RPE	retinal pigment epithelium
OS	outer segment
IS	inner segment
OMIM	Online Mendelian Inheritance
MSS	Mainzer–Saldino syndrome
SRTD	Short-rib thoracic dysplasia
ATD	Asphyxiating thoracic dystrophy
SRPS	Short-rib polydactyly syndrome
IFT	Intraflagellar ciliary transport
RPGR	Retinitis GTPase Regulator
RPGRIP1	RPGR-Interacting Protein 1
IQCB1	IQ calmodulin-binding motif-containing protein 1
JS	Joubert Syndrome
MKS	Meckel syndrome
BBS	Bardet–Biedl syndrome
OFD	Oral–facial–digital syndrome
ALMS	Alström Syndrome
USH	Usher syndrome
CNS	Central Nervous System
MTS	Molar Tooth Sign
DPM	Ductal plate malformation
AAV	Adeno-associated virus
